# Antireductionisms with regard to mental disorders: some caveats. A commentary on Marco Stier

**DOI:** 10.3389/fpsyg.2014.00350

**Published:** 2014-04-29

**Authors:** Bettina Schoene-Seifert

**Affiliations:** Institute for Ethics, History and Theory of Medicine, University of MünsterMünster, Germany

**Keywords:** mental disorders, non-reducibility thesis, medical model of mental disorder, psychiatric diagnostics, value-dependence

## Introduction

With his article “Normative preconditions for the assessment of mental disorder” Stier (2013) is presenting a thought-provoking piece of work and I agree with many of his conclusions. This is certainly true of Stier's main thesis that the demarcation line between mental health and mental disorder cannot plausibly be gained on the level of neurobiology alone, but is in need of additional value judgments. However, I think that this specific “antireductionist claim” holds true also in somatic medicine. Hence, the “medical model,” rightly understood, seems to be fully appropriate for assessing mental disorder. Moreover, I suggest to be very restrictive in discussing the concept of psychiatric disease in the language of reductionism, since this might, contrary to Stier's own intentions, be easily misunderstood as water on the mills of methodological antireductionism in psychiatry.

## Setting the stage

Making use of Ayala's (1974) influential differentiations between reductionism (and the corresponding debates) on the levels of metaphysics (ontology), epistemology, and methodology, reductionist concerns vis-à-vis psychiatry primarily refer to the last level. As a practical science, psychiatry is mainly concerned with *methods* or strategies of preventing, ailing, or curing mental disorders. These strategies in turn are interrelated with methods of properly explaining and diagnosing such disorders. In contrast, whatever psychiatrists or their critics hold on the level of ontology or epistemology seems relevant to psychiatric work (only) in so far as it determines outlooks on methodology—especially in interacting with patients and in treating their disorders. When it comes to the latter, matters of causation play the crucial role. And here, I urge, one should distinguish between two questions: (i) how mental dysfunctions (e.g., delusions, depression, mania, decrease in cognitive functions etc.) is/can at all be “caused” by brain dysfunction; (ii) how relevant systemic brain dysfunction is caused by neurobiological processes on lower levels—e.g., on the levels of circuits, cells, or genes.

The first question points to the central and perennial problem of the mind-brain debate and from here cuts throughout psychiatry (so also Kendler, 2008, p. 9). For these problems and questions, it ultimately does not matter whether we talk about healthy or disordered minds and brains. I do not know whether psychiatry might make a genuine contribution to solve these problems. Likewise, we most often do not know what proponents or critics of biological psychiatry hold in these matters. Beyond the shared views that the “mental realm,” disordered or healthy, is (a) both very real and very important to ourselves and (b) brain-based, there exist many conflicting views and intuitions. Key problems seem to be the questions of mental causation, agent causality, and free will. In this paper, Stier does not address them in their own right, but he suggests assuming full explicability of the mental in “purely physical terms” (p. 2).

The second question lies at the bottom of what mainstream neuroscience, and in fact life science in general, is doing today. Here, scientists successfully try reductionist strategies to count for certain biological phenomena by explaining them on a relatively lower level (circuits, nerve cells, synaptic spaces) and by isolating them from as many relevant background conditions as seems fruitful^1^. Here again, Stier is ready to accept—if only for the argument's sake—“that environmental influences, too, are explicable mechanistically” (p. 2). Making such (non-eliminative) reductionist assumptions upon both questions, he rightly emphasizes that the truth of his “anti-reductionist claim” regarding the notion of mental disease does not depend on metaphysical or methodological anti-reductionism with regard to the mental.

## A partially normative concept of (mental) disease

Stier holds that “mental disorders cannot be completely reduced to neuronal or molecular processes” (p. 1). His justification for this “anti-reductionist claim” is the above stated “main thesis” which holds that in the field of neuropsychiatric disorders, the borderline between health and disease is value-laden. Unable to argue for this in any detail, I wholeheartedly agree with the view that the concept of mental disorder is partly normative. Being mentally diseased means (or should mean) to be in some or other dysfunctional and unwelcome mental state that thus should ideally be prevented or treated. Imprecise as these stipulated evaluative criteria and their originators are, I also agree with the view that individual and social value judgments cannot be read off from mere neurobiological facts^2^. We principally cannot tell from scratch whether some functional neurobiological state corresponds to a mental disease or not. Rather, we can only do so within a partly evaluative background frame.

However, do these insights not hold true for diseases in *general*, for disorders within *and* without psychiatry? For so-called somatic disorders, this might not always be as obvious as in the realm of psychiatric diseases. Take an infection that, if untreated, would rapidly lead to death without any other adverse symptoms. One might argue that premature death is a purely descriptive term independent of it's being unwelcome to most people. But the same could be said about neuro-psychiatric disorders that lead to permanent coma or benign delusions. Where single disorders, in the mental as well as in the non-mental sphere, *seem* to be explicable without recourse to values, the gist of the whole concept of disease refers to unwelcome malfunctioning (including the functions of living or being conscious) and can be traced, I think, in each of its sub-types. Unable to further argue in favor of a partly normative concept of disease at this occasion, let me at least emphasize that this is one of the standard views (often referred to as partial “constructivism”) in the contested field of theories of health and disease (see Murphy, 2008). The current tendency to blurr or to give up the distinction between psychiatry and neurology could, by the way, be seen as yet another indicator for the non-exceptionalist status of mental disorders (see Perring, 2010).

## The medical model

Stier refers to the “medical model” (MM) without giving a complete explicit definition. In the literature, MM is indeed a commonly used paradigm; it is seen, however, to allow for “minimal and strong interpretations” (Murphy, 2010, pp. 3–13). Stier's understanding of MM comes in pieces. On a purely descriptive level it is said to stand in competition with psychoanalytical and other explanations of mental disorders (p. 7) and to substantially parallelize body-environment interaction in the genesis of cancer and brain-environment interaction in the genesis of depression (p. 2). Critically, MM is accused of inadequately explaining psychiatric disorders: “psychiatric disorders [… ] may turn out to be purely physical if we adhere to the medical model [… ] and cease to be mental” (p. 8). But why should this be the case?

One possible answer could be MM's alleged tie to a value-neutral concept of disease. However, this is not only contested by many and with good reasons (see above), but also by Stier himself. He clearly admits that “[… ] it is not that bodily diseases are value-free whereas psychiatric disorders are value-laden. Both rest on normative assumptions.” But then he continues: “In one field we simply share them, in the other we don't” (p. 5). Both observations of the last sentence seem questionable: Quite a number of “bodily” conditions are contested with regard to their “diseasedness”—e.g., limited reproductive or sexual functions, moderately decreased hearing, or moderately diminished memory capacities in “normal aging.” Arguably, it is normative aspects that will determine demarcations. In any case, MM does not seem committed either to value neutrality in the concept of disease, or to the indisputability of the underlying values. On the other hand, value dissent in the psychiatric domain is by no means ubiquitous. After all, delusions, anxiety disorders, depression, or addiction do not appear very attractive, neither from inside nor from outside.

Hence, contrary to Stier, MM should in my eyes be properly understood as *rightly* holding a thoroughgoing non-exceptionalist view toward the explicability of psychiatric disorders. This view indeed seems to be the main stream position in neuroscience. It implies optimism with regard to neuroscientific contributions to diagnostic and therapeutic progress in psychiatry. But, again, it does neither imply viewing the concept of psychiatric disorder as value-independent nor viewing the mental realm as eliminable by neurobiological approaches.

## Psychiatric diagnostics

Suppose, you diagnose an individual patient with certain symptoms as suffering from mental disorder Z. In an idealized nutshell this presupposes: (1) a multi-dimensional demarcation between mental sanity and mental diseasedness, where those symptoms indicate disorder; (2) a taxonomy of *specific* psychiatric diseases, one of them called Z; (3) valid *indicators* and tests for Z; (4) positive *testing* for indicators of Z in the concrete patient. Each of these steps has its problems. But only (1) seems value-dependent in the way described by Stier, i.e., relative to human flourishing and human interests. With regard to (2) there is malleability and ongoing change in both the bodily and the psychiatric dimension of medical practice: fine-tuning and re-tuning according to some symptoms or other, to locations, or to (assumed) underlying causal paths. The main values that reign nosology are coherence and therapeutic success, I think. (3) is, again, an ongoing process according to medical evidence, having repercussions to (2) and being reigned by the very same values of coherence and therapeutic effectiveness. Finally, diagnosing a given patient should involve testing her according to best available parameters, with results of presuppositions (1) to (3) in the back. Hence, in psychiatry, a patient showing up with certain behavioral symptoms could conceivably be tested for neurobiological indicators, resulting in the diagnosis Z—without loosing sight of the mental. Determining a mental disorder in this way is not guilty of any problematic reductionist credo.

## The inner life of psychiatric patients

Granting potential causal relevance to a multitude of external influences, psychiatrists would finally be ill advised to look for brain function in isolation rather than in context. But turning external effects—e.g., psychologically stressful life events—into background conditions of pathogenesis, does not imply neglecting their causal role. Nor does it imply ignoring the importance of preventing such adverse factors in the first place, or excluding psychotherapy from the agenda of psychiatry. Likewise, nothing in a methodologically reductionist approach to psychiatric research compels scientists or doctors to ignore or belittle the enormous importance of patients' conscious experiences. If such unfortunate “practical reductionisms” nevertheless occur, they can neither legitimately be nobilized nor criticized as a sequel of biological psychiatry.

From all we know and foresee, detailed knowledge about one's inner mental life needs first-person experience or, as a weak approximate substitute, third-person encounter. Listening to psychiatric patients' directly or indirectly describing their subjective experiences thus seems irreplaceable for assessing the subjective impact of mental disease as well as for an understanding interaction with patients. Nevertheless, using neurobiological tools for diagnosing and monitoring treatment might in principle be possible and helpful.

## Summing up

Stier holds that the classification of certain mental states as disorders is value-dependent and therefore cannot be read off from neurobiology. Contra Stier, however, this plausible view does in no regard discredit the medical model (MM) as “the one and only bedrock of psychiatry” (p. 1). Rather, MM is uncommitted to a naturalist theory of disease. As Stier himself admits, values can be seen as indispensible also in demarcating bodily diseasedness. Some of these diseases and values might be as contested as in psychiatry. MM's upshot is a non-exceptionalist view on the explicability of psychiatric disorders—and subsequently on their diagnostic and therapeutic in-principle accessibility on a biological level.

Finally, framing and selling a partially constructivist position regarding (mental) disease as an *anti-reductionist* view, is both unusual and misleading. Affirming such constructivism should not get confounded with common and problematic objections that blame biologically oriented psychiatry as metaphysically or methodologically reductionist. Yet another distinct problem might be an unfortunate practical negligence of patients' inner life within modern psychiatry. Such “practical reductionism” can and should be defeated *within* a neurobiological orientated psychiatry.

### Conflict of interest statement

The author declares that the research was conducted in the absence of any commercial or financial relationships that could be construed as a potential conflict of interest.

## Footnotes

^1^See Kaiser (2011) for a diligent analysis.

^2^See Barker and Kitcher (2014), p. 70ff. for a defense of value invention.
